# Trends in the numbers of SARS-CoV-2 infections among students: a prospective cohort study comparing students in sports boarding schools with students in day schools during early COVID-19 pandemic

**DOI:** 10.3389/fpubh.2023.1223748

**Published:** 2023-11-14

**Authors:** Friedrich Barsch, Vera Peters, Oliver Morath, Oliver Krumnau, Philipp Maier, Daniela Huzly, Stephan Prettin, Peter Deibert

**Affiliations:** ^1^Department of Medicine, Medical Center University of Freiburg, Faculty of Medicine, Institute for Exercise and Occupational Medicine, University of Freiburg, Freiburg im Breisgau, Germany; ^2^Freiburg University Medical Center, Faculty of Medicine, Institute of Virology, University of Freiburg, Freiburg im Breisgau, Germany

**Keywords:** COVID-19, SARS-CoV-2, athletes, students, schools, prevention, sports

## Abstract

**Introduction:**

During the first months of the COVID pandemic it emerged that facilities where people gather or live together in cohorts, such as nursing homes or schools, were particularly at high risk for becoming hotspots of virus transmission. German political and health institutions responded with far-reaching interventions and preventive strategies to protect the population from infection with SARS-CoV-2. In this context, it remains unclear whether boarding schools for sports particularly pose a risk of infection to their residents.

**Methods:**

In a single-center prospective cohort study, numbers of SARS-CoV-2 infections of students in sports boarding schools (*n* = 11) vs. students attending regular day schools (*n* = 22) in the region Freiburg/Hochschwarzwald in Germany were investigated over a period from October 2020 to January 2021 via regular virus and antibody screening (German Clinical Trials Register; Study ID: DRKS00021909). In addition, individual and behavioral risk factors for infection were stratified via questionnaire, which provide an indication of cohort specific risk factors for infection and the success of the implementation of hygiene concepts, as well as other infection prevention strategies, within the respective facilities.

**Results:**

Regarding SARS-CoV-2 infection numbers, the screening detected no significant group difference between sports boarding schools vs. day schools.

**Discussion:**

The study results provide indications that sports boarding schools did not pose an increased risk of infection, assuming that the facilities prevent virus transmissions with appropriate preventive strategies and hygiene measures. In future pandemic scenarios larger-scale and multicenter studies are necessary to achieve more comprehensive epidemiological data in this field.

## 1 Introduction

Since the beginning of 2020, the spread of SARS-CoV-2 has affected the lives of billions of people. First detected in Wuhan (Hubei, China) in December 2019, the pathogen spread mainly via aerosol transmission all around the world (1, 2). On March 11, 2020, the World Health Organization (WHO) classified the outbreak of SARS-CoV-2 infections as a pandemic and by that time 118,319 confirmed cases and 4,292 deaths from the disease had been reported worldwide. The novel pathogen also spread rapidly in Germany and soon it became evident that cohort-housed facilities in particular were at high risk for viral transmission (3). Faced with nationwide increasing infection rates and in the absence of sufficient vaccination rates, the German federal government and all 16 German state governments adopted far-reaching interventions and preventive strategies to protect the population from infection with SARS-CoV-2, which resulted in a general lockdown and closure of several public facilities. In this context, the state government of Baden-Württemberg in southwest Germany decided to close schools from March 17, 2020. In some cases, the school closures were maintained for several months, depending on regional incidence and grade levels. Consequently, the students' and adolescents' circumstances changed significantly and their new daily routine between homeschooling, complying with hygiene procedures and prevented extracurricular activities turned into a major challenge (4).

At the end of September 2020 a second wave of infections with SARS-CoV-2 occurred in Germany reaching its highest incidence level in December 2020 with up to 32,195 new daily infections on December 24, 2020. With the onset of the second COVID-19 wave another debate emerged in Germany as to whether school lockdowns were an appropriate instrument for containing the incidence of infection and whether the students could be expected to go through another school lockdown with all its challenges. According to the epidemiological bulletin of the German Robert Koch Institute, data on the infection environment of registered cases within Germany were published on August 11, 2020 (3). Here, infections in private households formed the largest proportion, followed by senior citizens and nursing homes as well as refugee and residential homes. The working space formed the second largest infection environment followed by shared residences (3). However, no robust data existed on how the infection distributed in the collective of students in schools and to what extent the infection numbers develop after reopening of schools. It was also unclear whether there were differences in certain types of schools, for example differences between boarding schools and regular day schools.

In this context, the Institute of Exercise and Occupational Medicine of the University Medical Center of Freiburg, Germany, aimed to provide information on whether attending sports boarding schools (SBS) poses an increased risk of infection with SARS-CoV-2 compared to regular day schools (DS), assuming that SBS students would be at higher risk for infection due to cohort accommodation and increased sport-related interpersonal contacts. In addition, the question arose as to whether a corresponding closure of SBS should occur in analogy to the day schools in terms of infection prevention. At the same time, possible individual and institutional risk factors for infection and resulting prevention measures were investigated. The study wanted to gain experiences and possible differences in the occurrence and transmission of infection with SARS-CoV-2 within a SBS or a DS. This may potentially prompt further cohort-specific information, which could lead to more sophisticated recommendations in an epidemic or pandemic situation for respective student facilities.

## 2 Materials and methods

### 2.1 Study design and procedure

A single-center, non-interventional, prospective cohort study was chosen as an appropriate observational study design. Two age-matched cohorts of students were observed regarding the occurrence of SARS-CoV-2 infections. Cohort 1 (SBS group) consisted of competitive sports students residing in sports boarding schools in the region Freiburg/Hochschwarzwald in southwest Germany. Cohort 2 (DS group) consisted of students attending regular day schools in the same region. The cohorts were observed in parallel over a period from October 2020 to January 2021. The chosen period corresponded to the second wave of COVID-19 diseases in Germany. The study was registered in the German Clinical Trials Register under the corresponding Study ID: DRKS00021909. After recruitment, informed consent, and study inclusion, subjects received a baseline visit (t0) and additional follow up examinations after 4 (±1) and 8 (±1) weeks (t1 and t2). A time buffer of ±1 week was tolerated to avoid scheduling conflicts and, if possible, to invite all study participants within a group together for follow-up investigations. Initial appointments were arranged individually with the students from the corresponding SBS and DS. Follow-up appointments were organized according to the defined interval starting from the timepoint of the first visit. In both cohorts, the respective diagnostic and survey procedures were applied equivalently (see Table 1). The primary outcome parameter was defined as the detection of SARS-CoV-2 infection at timepoints t0, t1, and t2. In addition, secondary endpoints were surveyed at each time point to gain insights into individual and institutional risk factors for infection. Thereby, the subjects were asked via interview and questionnaire about symptoms of illness, social contact activities in the private and sporting setting, exposures to infected individuals, travel behavior and compliance with hygiene regulations, preventive strategies and their subjectively assessed effectiveness. The sport boarding schools' own preventive and hygienic concepts for infection prophylaxis was provided by the participating institutions. Their preventive concept included, in addition to the generally applicable hygiene rules (wearing facemasks, adhere the distance rules and regular hand hygiene) a query of infection symptoms as well as an obligatory hand disinfection upon entry into the institution, a daily disinfection of potentially contaminated surfaces (e.g., door handles), a general prohibition of visits, the drafting of an individual daily schedule of the athletes, the relocation to single rooms, the recommendation to avoid direct physical contact and to maintain a minimum distance of 1.5 m, as well as a distance maintenance when serving meals. Furthermore, it was decided that athletes with symptoms of infection would have to be immediately quarantined in their single rooms until a medical consultation, including a decision on the further procedure, was made. The detection and diagnosis of a SARS-CoV-2 infection had to be reported immediately to the competent health authority, which had to decide on the further procedure, up to a temporary closure of the respective facility.

**Table 1:** Schedule of data collection and diagnostic procedures with respect to cohorts and study timepoints.

	**Cohort 1**			**Cohort 2**		
**Timepoints**	**t0**	**t1**	**t2**	**t0**	**t1**	**t2**
Time period	10/2020–11/2020	11/2020–12/2020	12/2020–01/2021	10/2020–11/2020	11/2020–12/2020	12/2020–01/2021
**Assessments/data collection and sampling**						
- Verification of written informed consent	X			X		
- Screening and study inclusion	X			X		
- Acquisition of master data	X			X		
- Survey of sports activity	X			X		
- Oropharyngeal swab	X	X	X	X	X	X
- Venous blood sampling	X	X	X	X	X	X
- Questionnaire	X	X	X	X	X	X
**Diagnostics**						
- RT-PCR	X	X	X	X	X	X
- ELISA based antibody diagnostics	X	(X)	X	X	(X)	X

### 2.2 Study population, recruitment and screening

**Figure 1:**
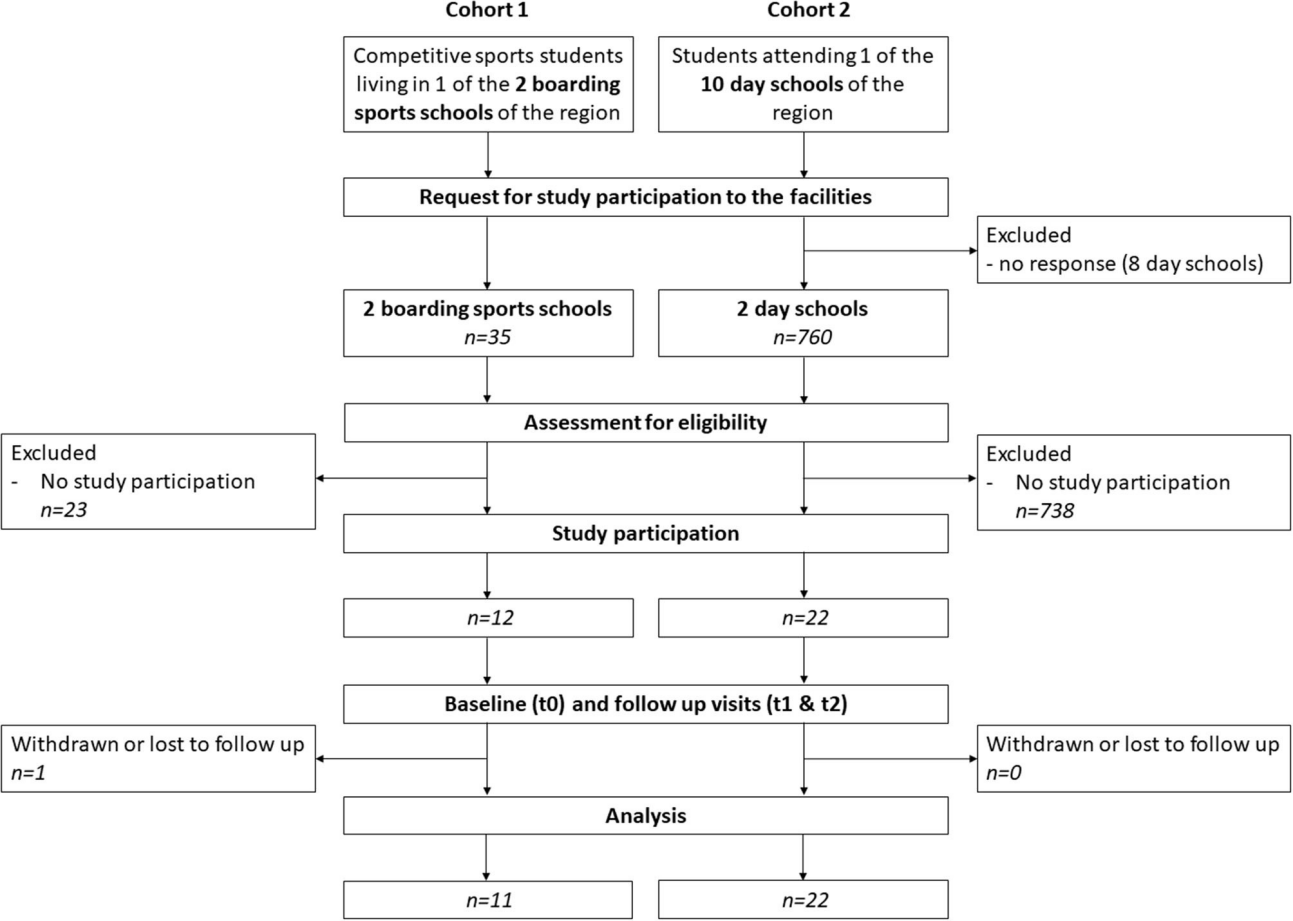
Study flow chart.

The study population consisted of students of a certain age (16–20 years old), who either lived in SBS (cohort 1) or attended DS (cohort 2) (see Figure 1). The inclusion and exclusion criteria are noted in Table 2. Students in cohort 1 (SBS group) were all competitive athletes, who spent most of their extracurricular time practicing different sports activities in their training groups. Due to the pandemic situation, a bubble principle was performed in the training groups in such a way that all members of a training group were instructed to avoid contacts in the non-sporting area as much as possible. Symptomatic athletes were not allowed to attend ongoing training sessions. Due to the profile of the SBS, these were primarily winter sports (cross-country skiing, biathlon, ski jumping), which are mostly practiced outdoors as individual sports, but were also exercised in training groups of several persons. On the weekends, SBS students usually were either at competitions or went home to visit their families. Beyond study participation athletes received PCR diagnostics regarding SARS-CoV-2 infection in case of symptoms and before and after competitions. Students in cohort 2 (DS group) lived at home with their families and spent their leisure time doing different activities. Beyond study participation and in accordance with the official regulations at the time, students in this cohort had to undergo PCR testing if they were symptomatic.

**Table 2:** Inclusion and exclusion criteria.

	**Cohort 1**	**Cohort 2**
Inclusion criteria	- Students residing in a sports boarding school	- Students attending a day school
	- Date of birth between 01. November 2000 and 01. November 2004	- Date of birth between 01. November 2000 and 01. November 2004
	- Absence of signs of infection (e.g., fever)	- Absence of signs of infection (e.g., fever)
	- Unrestricted suitability for sports	- Unrestricted suitability for sports
Exclusion criteria	- Known previously experienced SARS-CoV-2 infection.	- Known previously experienced SARS-CoV-2 infection.
		- Participation in a club sport according to the ACSM (“American College of Sports Medicine”) of more than 2 times per week.

In a preliminary step, potential institutions in the region were screened via web-based research. In this process, ten DS and two SBS could be identified for possible study participation. Subsequently, heads and administrations of the respective institutions were informed in written form about the planned study project and asked about the possibility of conducting the study within the institution. The request included an offer for a study information session for teachers, students and parents at the respective facilities. According to the guidelines of the Declaration of Helsinki, participation in the study was explicitly advertised as voluntary. With this strategy, two SBS and two DS could be won for study participation. In case of positive feedback from the facilities, a verbal and written study information was provided for interested students and parents. In order to avoid unnecessary contacts of the study personnel with the institutions, responsible teachers/guardians in the institutions took over the information transfer about the study project. Prospective students who matched the age range were invited to participate in the information session with the study personnel at the respective facilities. Whenever there was an entry of study personnel into the facilities, the study personnel excluded an own COVID-19 infection by PCR testing in advance and entered the facilities only in appropriate protective clothing in order not to cause virus transmission into the facilities. In a next step, interested students were screened according to inclusion and exclusion criteria (see Table 2). Only students who met the inclusion criteria and had given written informed consent to participate in the study were included. For minors, the signature of a legal guardian was obligatory. Due to pre-selection through the school's internal procedures and without the finding of other exclusion criteria, no prospective student had to be excluded in the screening process.

### 2.3 Data collection and data management

Data collection was either conducted at the study center or at the respective facilities. In this case, a mobile study team headed by a physician from the study center was sent to perform the data collection and diagnostics for the corresponding visits (see Table 1) according to the transmission prevention measures described in Study population, recruitment and screening. Analog case report files (CRFs) were used to record and save the subjects' master data (date of birth, gender, body weight in kg and height in cm), health-related data (previous illnesses, smoking habits) and study-specific data and documents (results from SARS-CoV-2 screening via RT-PCR, SARS-CoV-2 antibody diagnostics, paper-based questionnaire). CRFs were reviewed for legibility, completeness, and consistency via an internal monitoring at the study center.

After study inclusion, each study participant received a specific three-digit ID for pseudonymization. By means of two subsequent numerical digits, the collected biospecimens could be coded in a timepoint-related manner, which ensured pseudonymization to the sample processing Institute of Virology at the University Hospital Freiburg. Only the study director was given the authority to decipher the personal data to contact the appropriate local health department if a positive sample was detected. All other persons involved in the further processing of the specimens and data were only granted pseudonymized access to the data. The pseudonymized data were transferred to an electronic database for statistical analysis.

#### 2.3.1 SARS-CoV-2 virus diagnostics

Oropharyngeal swabs (Sigma Virocult^®^ swab kit, Medical Wire&Equipment Corsham, Wiltshire, SN13 9RT, UK, REFMW950S) were taken at each time point of the study for direct detection of infection with SARS-CoV-2. Realtime-PCR (RT-PCR) analyses of the swabs were performed at the Institute of Virology of the University Hospital Freiburg using the AltoStar^®^ SARS-CoV-2 RT-PCR Kit 1.5. The detection limit for SARS-CoV-2 in UTM^®^-containing simulated nasal matrix was 0.014 PFU/ml (95% confidence interval: 0.008–0.032 PFU/m) according to the manufacturer's instructions.

#### 2.3.2 SARS-CoV-2 antibody diagnostics

Venous blood sampling from the cubital vein was performed at each visit for indirect detection of an acute or past SARS-CoV-2 infection via antibody diagnostics. After storage on ice, centrifugation (Eppendorf brand centrifuge, model 5810 R for 15 min at 3,000 rpm), pipetting of the serum supernatant, its alliquoting and biobanking in−28°C (temperature-controlled Liebherr brand freezer), antibody analyses were performed at the Institute of Virology of the University Hospital Freiburg. All serum samples were analyzed at the end of the study using SIEMENS brand ADVIA Centaur COV2T assay. Total SARS-CoV-2 antibodies (including IgM and IgG) were evaluated. Samples with an index of <1 were considered negative for SARS-CoV-2 antibodies. Samples with an index >1 were considered positive for SARS-CoV-2 antibodies. The measurement range of the test was an index value of 0.05–10.00. According to the manufacturer, the sensitivity of the ADVIA Centaur COV2T test is 97.5% 7–13 days after a positive PCR test and 100% after 14 days. The specificity is 99.8%. In order to save analysis capacity and costs, serum samples from t0 and t2 were analyzed primarily. Only if the serum sample from t2 was positive for SARS-CoV-2 antibodies, serum sample from t1 was further analyzed in order to draw conclusions about the timepoint of infection if it were unclear. If positive antibodies had been detected at timepoint t0, this would have led to retrospective exclusion from the study and further analysis according to the exclusion criteria.

#### 2.3.3 Questionnaire

At all timepoints a questionnaire had to be completed by the study participants. The questionnaire was designed explicitly for the actual students' living conditions to assess an individual and institutional risk profile for SARS-CoV-2 infection. It considered topics and risk factors for SARS-CoV-2 infection, such as COVID-19 related symptoms, exposure to other persons in the sporting and family setting, out-of-school activities, adherence to hygiene regimens and travel behavior. The original questionnaire with its answer options and corresponding scale levels can be found in detail in Table 3.

**Table 3:** Questionnaire for the assessment of individual risk factors for SARS-CoV-2 infection of students in sports boarding schools and day schools.

**Topic**	**Questions**	**Answer options**	**Scale level**
Symptoms	Did you have symptoms of illness in the last 4 weeks?	Yes, no	Nominal
	- Which symptoms occurred?	No symptoms, fever, rhinorrhea, cough, sore throat, taste or smell disorders, headaches, earaches, dizziness, diarrhea, nausea/vomiting, other (free answer option)	Nominal
Meetings with friends	How often did you meet with friends outside the facility for recreational activities in the last 4 weeks?	Not at all, < 5 times, < 10 times, >10 times	Ordinal/Interval
	- Did you wear a facemask when you met your friends?	Yes, partially, no	Nominal
	- Did your friends wear a facemask during the meeting?	Yes, partially, no	Nominal
	- Did you keep the minimum distance of 1.5 meters during the meetings?	Yes, partially, no	Nominal
	- Where did you meet with your friends?	Indoor, outdoor	Nominal
Out-of-school activities	How often have you met with friends for sport sessions in the last 4 weeks?	Not at all, < 5 times, < 10 times, >10 times	Ordinal/Interval
	- What was the maximum number of persons you were at the sport sessions?	Number of persons	Rating
	- How many hours per week did you spend on sport sessions?	Number of hours	Rating
	Do you engage in regular after-school activities apart from sports (e.g., driving school, music lessons)?	Yes, no	Nominal
	- How many hours per week?	Number of hours per week	Rating
Public transport and travel behavior	How often have you used public transportation in the last 4 weeks?	Not at all, < 5 times, < 10 times, >10 times	Ordinal/Interval
	Have you been traveling in the last 4 weeks?	Yes, no	Nominal
	- If yes, how many days in total?	Number of days	Rating
Subjective assessment of hygiene measures	In your opinion, are the hygiene and distance rules (frequent hand hygiene, minimum distance of 1.5 m, wearing a facemask) being observed within the facility?	No, rarely, usually, always	Ordinal
	How useful do you consider the current pandemic-related restrictions?	Not at all useful to very useful (0–10 cm)	Rating

### 2.4 Statistical analysis

Statistical analyses were performed using SPSS Statistics 29 software (IBM, Armonk, New York). Data sets were examined regarding normal distribution using Shapiro-Wilk test. Nonparametric tests were used for calculation of statistical relationships. Exploratory statistical analyses were performed in dependence of group comparisons or the observation between different study time points. Mann-Whitney-U test (MWU) was applied for at least ordinally scaled, unrelated variables. Chi square test (χ^2^) or Fisher's Exact Test were applied for unrelated categorical scale samples. Cochranes Q or Friedmans test were used for connected samples. Furthermore, secondary outcome parameters were analyzed descriptively. Percentages were calculated to one decimal. All reported *p*-values are exploratory in nature. Significance level was set with *p* < 0.05.

### 2.5 Ethics approval statement

We adhered to the ethical principles of the World Medical Association's Declaration of Helsinki in the preparation of the study design as well as in the conduct of the study. An ethics application was submitted for the study, which was approved on 12/05/2020 (application no. 280/20). The study was reviewed and approved by: Research Ethics Committee, University of Freiburg, 79106 Freiburg, Germany. The students of full age provided their written informed consent to participate in this study. For all underage students, parents or guardians provided their written informed consent for their children to participate in the study.

## 3 Results

In the study region of the city and district of Freiburg/Hochschwarzwald two SBSs and two DSs showed interest in participating in the study. After screening of inclusion and exclusion criteria and verification of written informed consent, 12 students could be included in cohort 1 (SBS group) and 22 students in cohort 2 (DS group). During the study period one lost to follow up case was recorded in cohort 1 between timepoint t1 and t2 due to scheduling conflicts, which was excluded from statistical analysis. In cohort 2, all 22 study participants could be observed over the entire duration of the study.

### 3.1 Study population demographics

The study population consisted of two age-matched groups. The mean age of the participants was 17 years on average. Cohort 1 (SBS group) differed significantly from cohort 2 (DS group) in the characteristics of gender distribution, body height and weight and the weekly performed physical activity. No significant differences were found in relation to previous illnesses, usage of regular medication, smoking behavior, the number of family members or number of high-risk patients in the families (see Table 4).

**Table 4:** Demography of the study population.

**Parameter**	**Cohort 1**	**Cohort 2**	**Significance**
Age (years)	17.45 (±2)	17.09 (±1)	0.955
Gender			0.024^*^
- Male	*n* = 8 (72.7%)	*n* = 6 (27.3%)	
- Female	*n* = 3 (27.3%)	*n* = 16 (72.7%)	
- Divers	*n* = 0	*n* = 0	
Body height (cm)	181.78 (±9.79)	171.39 (±9.76)	0.011^*^
Body weight (kg)	70.58 (±8.67)	62.25 (±9.19)	0.024^*^
At least one pre-existing illness (yes)	*n* = 1 (9.1%)	*n* = 8 (36.4%)	0.212
Regular medication (yes)	*n* = 1 (9.1%)	*n* = 4 (18.2%)	0.643
Smoking (yes)	*n* = 0 (0%)	*n* = 1 (5%)	1.000
Family members (number)	3.54 (±1.3)	3.18 (±0.9)	0.560
At-risk patients in family (yes)	*n* = 10 (90.9%)	*n* = 17 (77.3%)	0.637
Sports activity (hours/week)	13.0 (± 4.2)	3.3 (± 2.4)	< 0.001^*^

^*^Significance level *p* < 0.05.

### 3.2 SARS-CoV-2 infections during the observation period

Results of detected SARS-CoV-2 infections in the respective cohort are shown in Table 5. A total of 33 oropharyngeal swab specimen and 33 venous blood samples could be obtained over the entire study period in the SBS group (cohort 1) for RT-PCR and antibody analyses. In the DS group (cohort 2), 66 oropharyngeal swab specimen and 66 venous blood samples were obtained. According to the diagnostics performed, only one positive SARS-CoV-2 infection could be detected in the SBS group by RT-PCR at timepoint t1, which could be confirmed by ELISA-based antibody diagnostics at timepoint t2. In contrast, no evidence of infection was detected in the DS group throughout the study period. In this context, the performed antibody analyses could not reveal any other hidden infection. Thus, with respect to the numbers of SARS-CoV-2 infections, no statistically significant difference could be determined between the study cohorts (Fisher's Exact Test, *p* = 0.333, ϕ = 0.143).

**Table 5:** Results of primary outcome parameters throughout the entire study period.

	**Cohort 1/ SBS group (*n* = 11)**	**Cohort 2/ DS group (*n* = 22)**	***p-*Value**
SARS-CoV-2 infections	*n* = 1	*n* = 0	0.333^*^
•Positive RT-PCR	*n* = 1	*n* = 0	
•Positive antibodies	*n* = 1	*n* = 0	

^*^Significance level *p* < 0.05.

### 3.3 Symptoms of disease

**Figure 2:**
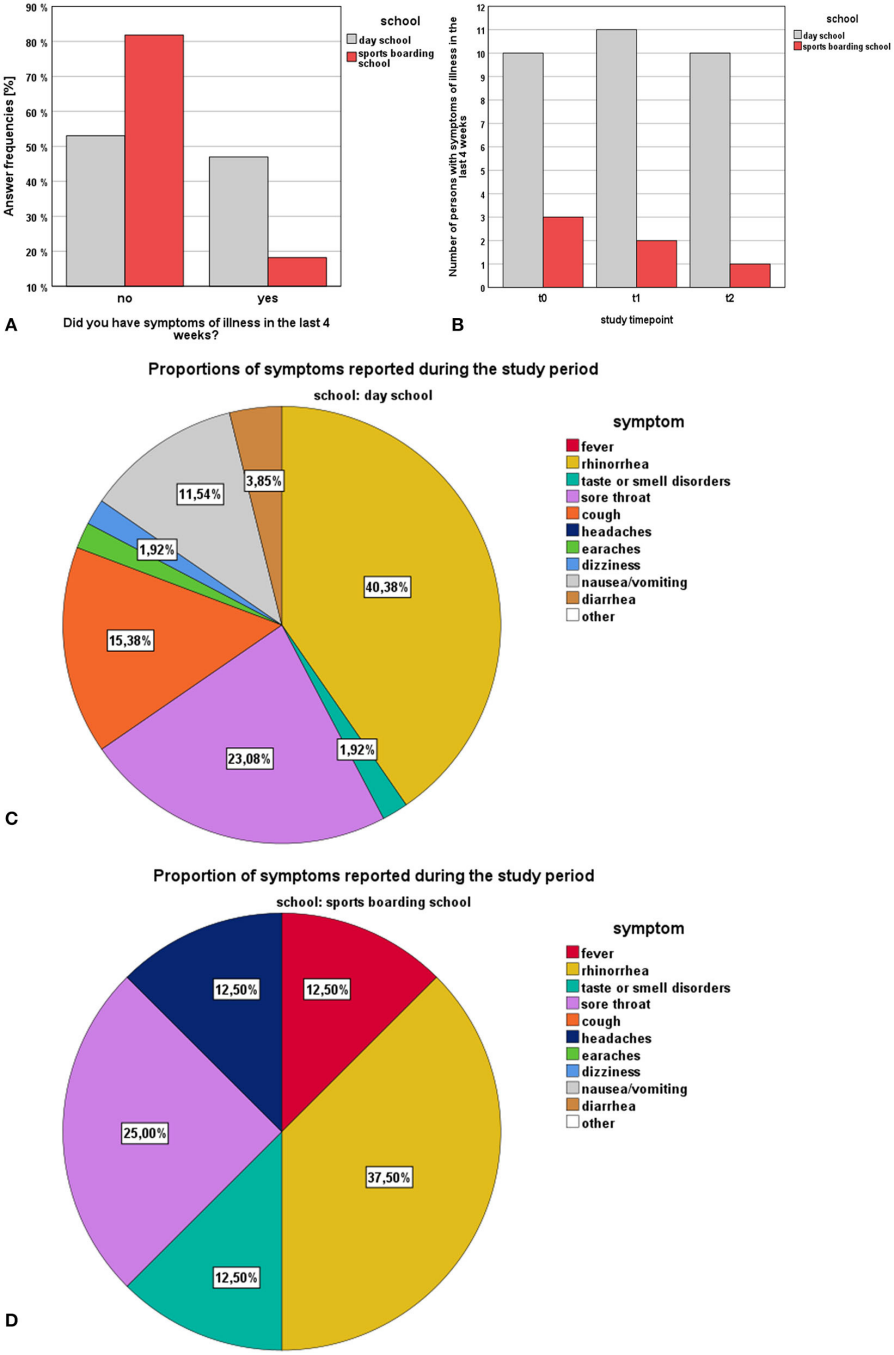
Survey results (SBS vs. DS) regarding the topic “symptoms”: **(A)** answer frequencies of SBS and DS students regarding the general occurrence of symptoms, **(B)** number of persons with symptoms during the last 4 weeks stratified by timepoints, **(C)** proportional distribution of symptoms encountered in DS students, **(D)** proportional distribution of symptoms encountered in SBS students.

Survey results are presented graphically in Figure 2 and in tabular form in Table 6. Considered the entire study period Fisher's Exact Test showed no significant difference in the occurrence of symptoms of illness between the study groups (*p* > 0.05). A closer look across the temporal course of the study showed a timepoint related decrease in the occurrence of one or more symptoms during the last 4 weeks in the SBS group [27.3% (t0) vs. 18.2% (t1) vs. 9.1% (t2)], while in the DS group the occurrence of one or more symptoms during the last 4 weeks remained fairly equal [45.5% (t0) vs. 50.0% (t1) vs. 45.5% (t2)]. But, Chochran's Q Test did not reveal a statistical significant difference in the data distribution between the timepoints within the respective study cohorts.

**Table 6:** Survey results on the occurrence of symptoms in the last 4 weeks according to study time points.

	**Cohort 1/SBS group (*****n*** = **11)**			**Cohort 2/DS group (*****n*** = **22)**		
**Timepoints**	**t0**	**t1**	**t2**	**t0**	**t1**	**t2**
**Symptoms**						
Fever	-	*n* = 1	-	-	-	-
Rhinorrhea	*n* = 1	*n* = 1	*n* = 1	*n* = 9	*n* = 7	*n* = 5
Taste or smell disorders	-	*n* = 1	-	*n* = 1	-	-
Sore throat	*n* = 2	-	-	n = 5	*n* = 4	*n* = 3
Cough	-	-	-	*n* = 4	*n* = 3	*n* = 1
Headaches	-	*n* = 1	-	-	-	-
Nausea/vomiting	-	-	-	*n* = 2	*n* = 3	*n* = 1
Diarrhea	-	-	-	-	*n* = 1	*n* = 1
Dizziness	-	-	-	-	-	*n* = 1
Earaches	-	-	-	-	-	*n* = 1

### 3.4 Survey results evaluating risk factors for infection

#### 3.4.1 Expositions and compliance with hygiene measures in meetings with friends and out-of-school activities

**Figure 3:**
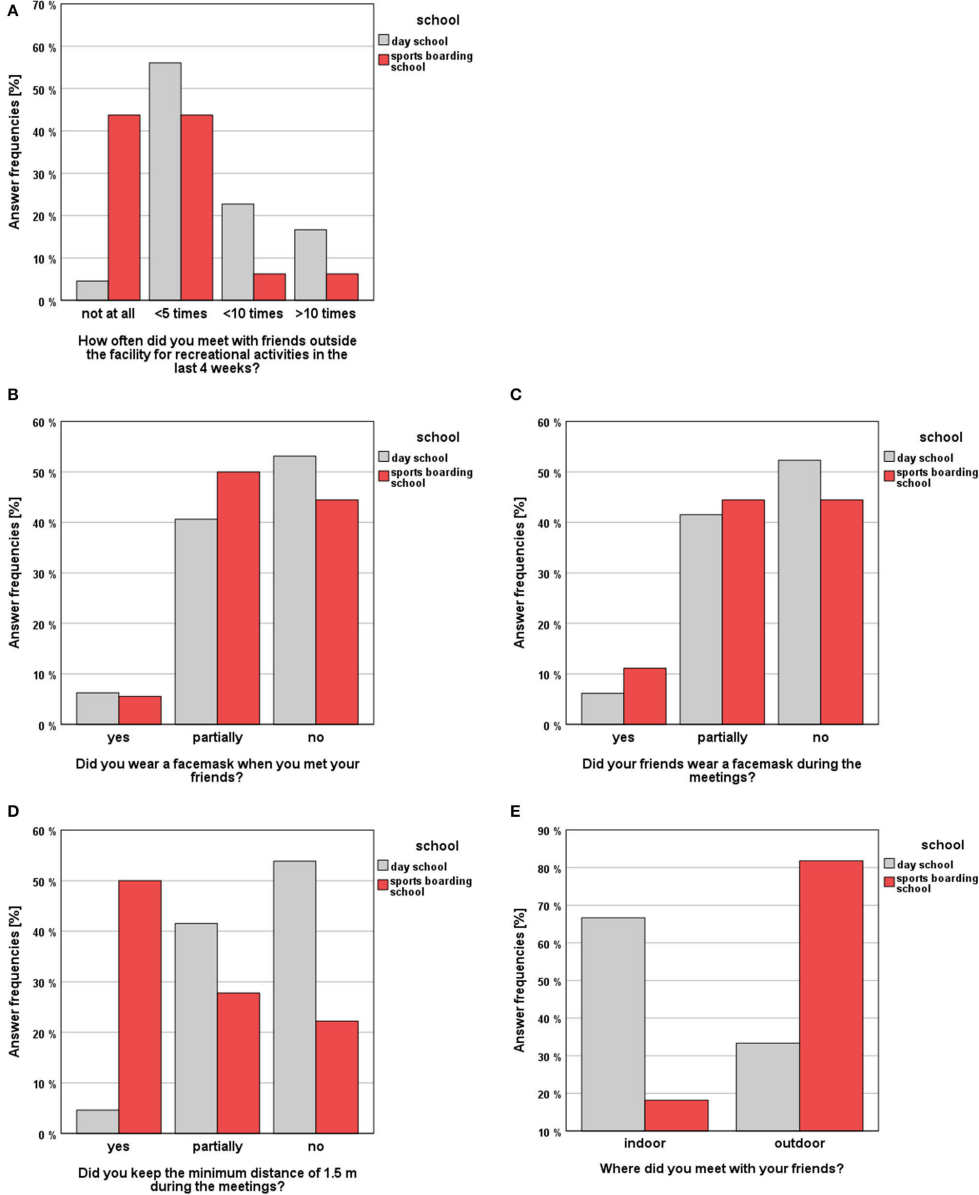
Survey results (SBS vs. DS) regarding the topic “Meetings with friends”: **(A)** answer frequencies regarding the frequency of meetings with friends, **(B)** answer frequencies regarding the wearing of facemasks of the students **(C)** and their friends, **(D)** answer frequencies regarding the compliance with minimum distance rules, **(E)** answer frequencies regarding the place of meetings.

**Figure 4:**
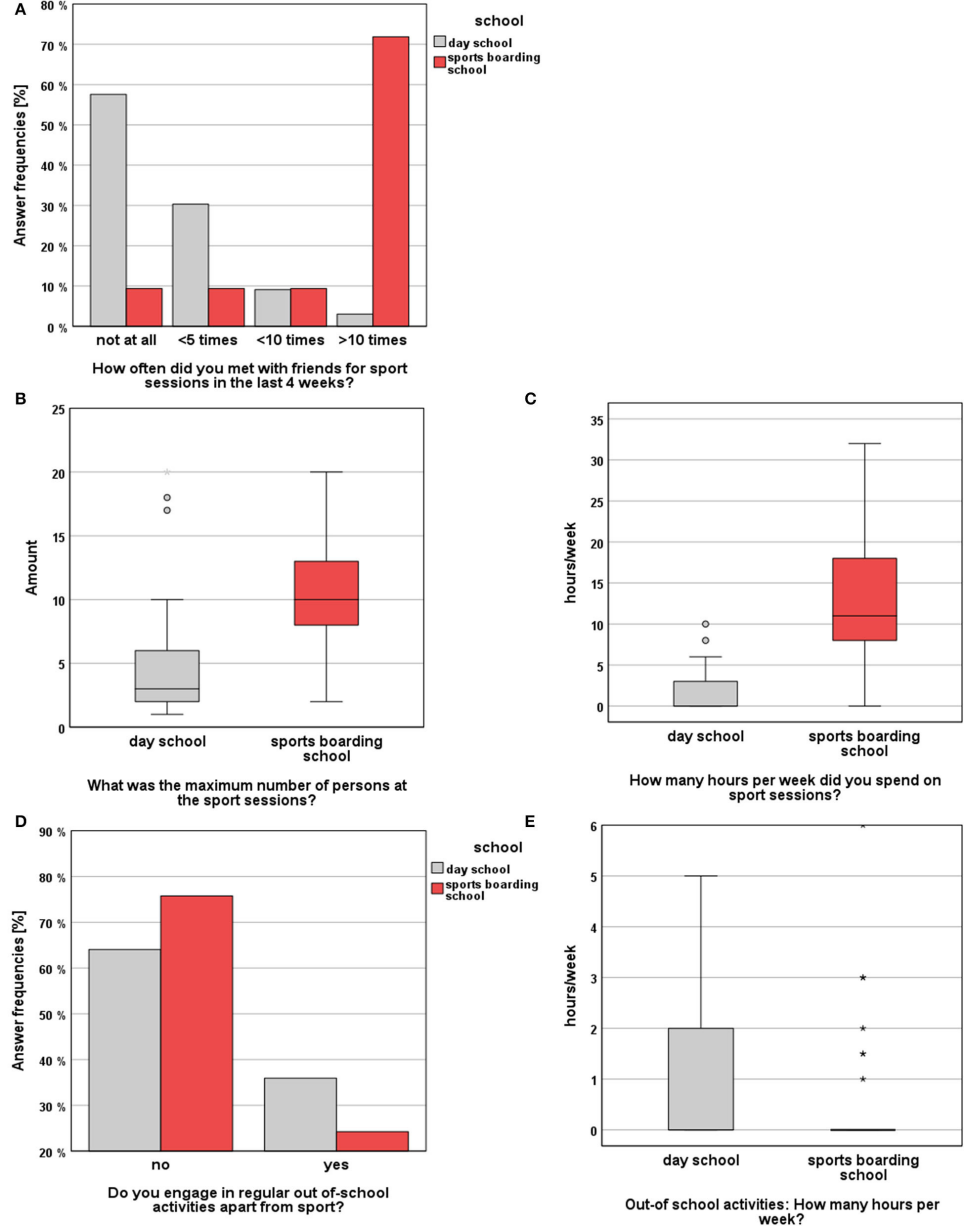
Survey results (SBS vs. DS) regarding the topics “Out-of-school activities”: **(A)** answer frequencies concerning the frequency of meetings for sport sessions, **(B)** maximum number of persons during sport sessions, **(C)** hours spent for sport sessions per week, **(D)** answer frequencies regarding the participation in out-of-school activities apart from sport, **(E)** hours spent for out-of-school activities per week.

Survey results are presented graphically in Figures 3, 4. Regarding the number of meetings with friends for recreational activities in the last 4 weeks before the respective visits, DS students were significantly more likely to meet with friends than SBS students (MWU, *p* < 0.001, *r* = 0.43). In this context, the frequency of meetings with friends showed a significant difference among the distributions between the study timepoints ($\chi {{}^{\text{2}}}_{\left(2\right)}$ = 11.902, *p* = 0.003). Here Friedman's pairwise tests revealed a significant decrease of meetings in the DS group between t0 and t2 (*p* = 0.035). With regard to the place of meetings, SBS students met friends significantly more often outdoor than indoor ($\chi {{}^{\text{2}}}_{\left(1\right)}$ = 6.428, *p* = 0.011, ϕ = 0.471). Regarding the wearing of a facemask in the context of the meetings, there were no significant differences between the study groups, with respect to the students and their met friends. In the SBS group, athletes wore a facemask permanently in only 5.6% of the cases and no mask was worn in 44.4% of the cases. In the DS group it was stated in 6.3% of the cases that a facemask was worn and in 53.1% no facemask was worn during the meetings. With respect to the adherence to the general rule of recommended minimum meeting distance of 1.5 m, there was a significant difference between the study cohorts. (Fish-er-Freeman-Halton Exact Test = 19.137, *p* < 0.001, ϕ = 0.535). SBS students kept the minimum distance in 50.0% of the cases, whereas DS students kept the distance in only 4.6% of the cases.

With regard to the performed sport sessions, SBS students met significantly more often with other people than DS students (MWU, *p* < 0.001, *r* = 0.66). A closer examination of the groups across the study time points indicated a significant change in the frequency of meetings for sport sessions within the DS group ($\chi {{}^{\text{2}}}_{\left(2\right)}$ = 17.098, *p* < 0.001). Here, Friedman's pairwise comparisons showed a significant decrease of the frequency of meeting for sports sessions between t0 and t1 (*p* = 0.004) and t0 and t2 (*p* = 0.010). On top, athletes met significantly longer with friends for sport sessions than DS students (MWU, *p* < 0.001, *r* = 0.70). In this context, SBS students spent an average of 12.7 h per week training with friends, whereas DS students met with others for an average of only 1.6 h per week for sports sessions. In terms of the number of training partners during exercise sessions, there was a significant difference between both study cohorts (MWU, *p* < 0.001, *r* = 0.47). In this regard, athletes met with a median of 10 other individuals. In contrast, DS students met with a median of three other persons. According to the place of the sport sessions both study groups showed no significant difference between indoor vs. outdoor locations (Fisher's Exact Test, *p* > 0.05). Regarding the practice of other out-of-school activities, such as music sessions, driving lessons, part-time jobs or church activities, no significant difference between both groups could be observed, both in terms of activities performed at all ($\chi {{}^{\text{2}}}_{\left(1\right)}$ = 1.370, *p* > 0.05) and the number of hours spent for activities (MWU, *p* > 0.05).

#### 3.4.2 Use of public transportation and travel behavior

**Figure 5:**
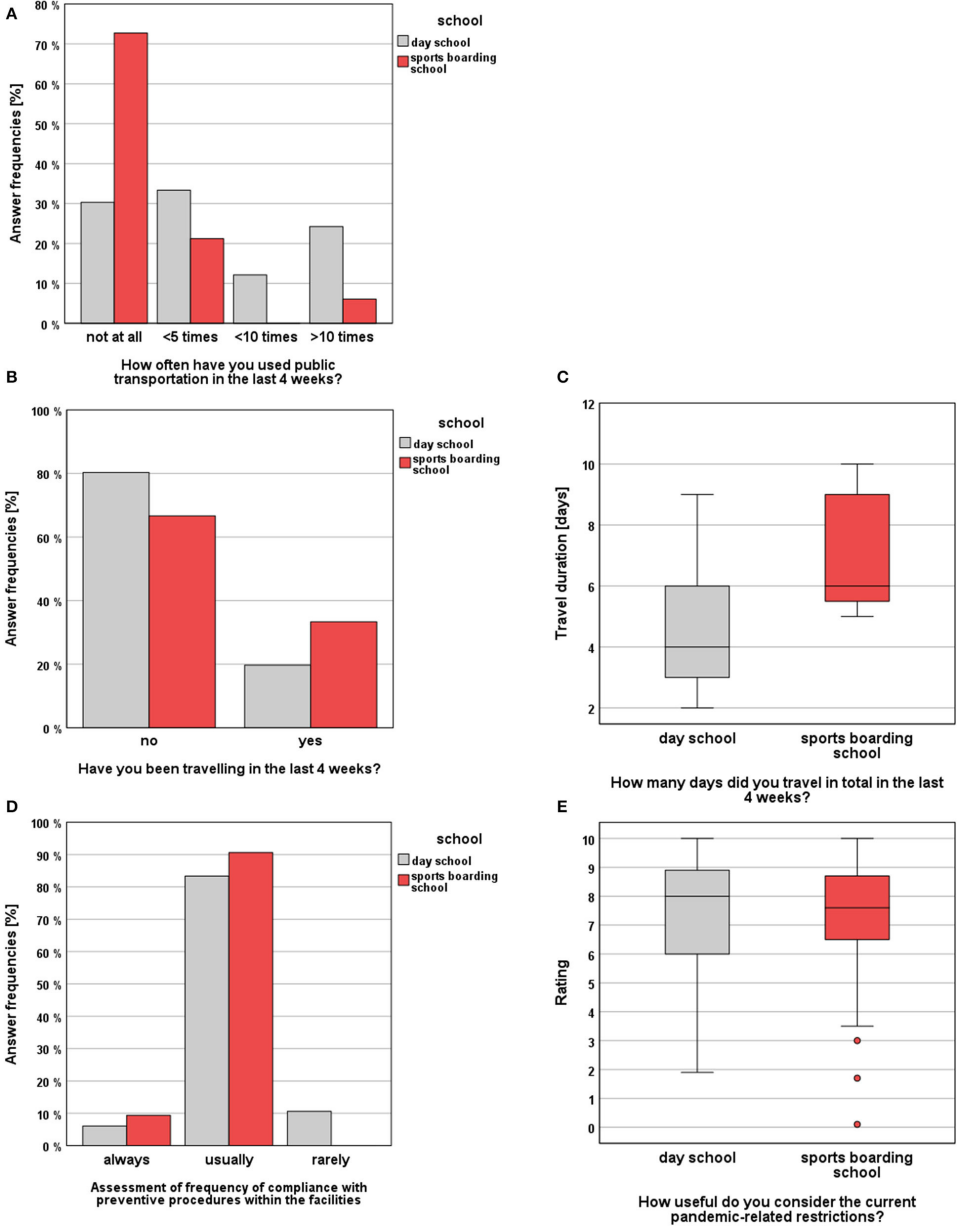
Survey results (SBS vs. DS) regarding the topics “Public transport and travel behavior” and “Subjective assessment of hygiene measures”: **(A)** answer frequencies regarding the frequency of public transportation uses, **(B)** answer frequencies regarding travels, **(C)** and travel durations, **(D)** students' evaluations of their institution's compliance with hygiene measures, **(E)** students' subjective assessments of the usefulness of the measures.

Survey results are presented graphically in Figure 5. In daily life, DS students used public transportation significantly more often than SBS students (MWU, *p* < 0.001, *r* = 0.41). They stated that they had “not at all” used public transport in the last 4 weeks before the visits in 30.3% of the cases (< 5 times: 33.3%, < 10 times: 12.1%, >10 times: 24.2%). In the SBS group, in 72.7% of the cases students stated that they had “not at all” used public transport in the last 4 weeks before the visits (< 5 times: 21.2%, < 10 times: 0.0%, >10 times: 6.1%). Regarding the overall performed travels during the entire study period no significant difference between the groups could be evaluated ($\chi {{}^{\text{2}}}_{\left(1\right)}$ = 2.228, *p* > 0.05). With respect to the last study timepoint (t2), a significant difference between the groups could be revealed. Here, athletes traveled significantly more often in the last 4 weeks (Fisher's Exact Test *p* = 0.030, ϕ = 0.447). Furthermore, it was noticed that the frequency of travels in the DS cohort decreased significantly in comparison between the timepoints (Cochranes Q, *p* < 0.001). Here a significant decrease between t0 and t1 (*p* = 0.001) as well as between t1 and t2 (*p* < 0.001) could be observed. In the SBS group, there were no significant differences between the study timepoints (Cochranes Q test, *p* > 0.05). In terms of the median travel duration, a significant difference between the study cohorts could be revealed (MWU, *p* = 0.018, *r* = 0.48). Here SBS students traveled longer (6 days) compared to DS students (4 days).

#### 3.4.3 Subjective assessment of hygiene measures

Survey results are presented graphically in Figure 5. With regard to the subjective assessment of the usefulness of the applicable preventive and hygiene measures, no significant difference could be observed in between the study groups (MWU, *p* > 0.05). On a scale from 0 = “not useful” to 10 = “very useful,” SBS students rated the usefulness of the interventions with a median of 7.6 points and DS students rated with a median of 8.0 points. Looking at the students' assessments of the extent to which hygiene measures were adhered to in their facilities, no significant difference between the groups could be shown (MWU, *p* > 0.05). In the SBS group only 9.4% of the athletes stated that the hygiene rules are “always” followed (90.6% “usually,” 0.0% “rarely”), whereas in the DS group only 6.1% of the students stated, that the rules are “always” followed (83.3% “usually,” 10.6% “rarely”).

## 4 Discussion

The study primarily investigated the number of SARS-CoV-2 infections in students of two age-matched cohorts during the second wave of COVID-19 diseases in Germany, a time when widespread vaccination was not yet available and the pandemic had to be combated primarily by conservative hygiene measures and far reaching preventive strategies (e.g., lockdowns). During the study period, regular testing of students was not yet scheduled. For students the official pandemic recommendations at the time were to stay away from school if they showed symptoms of the disease and to undergo a PCR test performed at accredited centers. If the diagnostic test was negative, students could return to school. If SARS-CoV-2 infection was confirmed, students would have been quarantined at home for at least 10 days. The detected SARS-CoV-2 infections had to be reported to the regional health department, which decided when to end the quarantine. In this context, we asked for the registered infection numbers of all 16–20 year-olds in the city and district of Freiburg/Hochschwarzwald (*n* = 25.501) recorded by the public health department. In this age cohort, 549 new cases were recorded during the study period. In addition, the health office recorded infection outbreaks at schools in the region. Here, a total of 30 infections were recorded at 7 different schools during the study period.

In this context, the present study evaluated for the first time, based on infection numbers, whether or not living in a sports boarding school-a facility in the sense of a cohort accommodation-turned out to be an additional risk factor for infection with SARS-CoV-2. To the best of our knowledge, it is the only study that investigated the spread of SARS-CoV-2 in this kind of a student concerning setting. Due to insufficient evidence of how SARS-CoV-2 infections behaved in student collectives during the early time of the COVID-19 pandemic the study attempted to contribute and to improve our understanding of the institutional role of day schools and sports boarding schools in the pandemic situation. Furthermore, the investigation considered the behavior of students in their everyday life, so that cohort-specific risk stratifications could be derived. The study compared SBS students with students in regular day schools. With regard to the recorded epidemiological data, it can be determined that the study groups did not differ significantly in terms of age, preexisting illnesses, smoking behavior, family members or at-risk patients in the family, what allowed comparability. However, there was a disparity between the groups in terms of gender distribution and anthropometric data (body weight, body height). In this context, no evidence could be found that body height influences the risk of becoming infected with SARS-CoV-2. But, Vahidy et al. (5) showed that males have an increased risk of testing positive for SARS-CoV-2 compared with females. Furthermore, there is evidence that male gender indicates a risk factor for more severe disease progression with SARS-CoV-2 (6, 7). Within the present study, the SBS group showed an increased proportion of male subjects, but due to the small number of participants, a gender-related risk for infection could not be drawn. Studies with a larger number of subjects would be necessary in order to be able to draw further conclusions here. With regard to body weight, an increased risk for severe courses of COVID-19 diseases has been shown, but there is no sufficient evidence that overweight also increases the probability of infection (8).

However, to ensure objective detection of SARS-CoV-2 cases, RT-PCR was used, which represents the gold standard for the diagnosis of SARS-CoV-2 infection (9, 10). For motivational reasons, and to avoid discouraging potential study participants, oropharyngeal swabs were chosen as an adequate sampling method because they were more tolerable to the students than nasopharyngeal swabs. Although some data suggest that oropharyngeal swabs have lower sensitivity than nasopharyngeal swabs, the argument of better tolerance was considered more important to improve the willingness to participate in the study (11, 12). In addition, both types of sample collection are considered to deliver adequate results (13). By obtaining venous blood samples for antibody diagnostics, it was possible to objectively record any asymptomatic infections that had occurred. Thus, it was possible to ensure recording of hidden infections, which were not detected by RT-PCR diagnostics, as it also could be shown by Buntinx et al. (9) in the setting of a nursing home.

In comparing SBS students with DS students, no significant difference in infection numbers (objectified by RT-PCR and antibody diagnostics) was found. Overall, infection counts remained very low in both groups. During the entire study period, only one SARS-CoV-2 positive case presented in the SBS group. In this cohort, it could have been assumed, that further (secondary) infections would occur due to cohort-like accommodation and increased interpersonal contacts during sports activities, for example. The cohort-like coexistence of athletes within SBS with shared use of living and dining areas could additionally be considered a particular risk factor for infection or viral transmission. This form of cohabitation can be compared to a larger household. According to several studies, most infections occur in the domestic setting, which could argue for an increased risk of secondary infections within the SBS cohort (14–16). Presumably, due to consistent implementation of hygiene and isolation measures and the consequent application of the bubble principle in the training groups, further spread of infections within the institution could be prevented. According to the hygiene rules of the SBS, the only infected person went into quarantine immediately after the onset of symptoms. Starting from this case and on the basis of the collected study data, no other infection occurred in study participants from the same sports boarding school. According to the information provided by the responsible sports boarding school director the other residents were also spared. Thus, it can be concluded that the hygiene concept and bubble principle, developed and implemented by the SBS management and coaches, was effective regarding the control of an in-house virus spread. The immediate performed quarantine seemed to prevent further SARS-CoV-2 infections, which was also shown by Li et al. (17) who could demonstrate in 105 index patients that an immediate implementation of a quarantine for an infected individual with symptom onset resulted in 0.0% secondary infections within the household compared to 16.3% without quarantine. However, the role of secondary infections in the school setting is discussed controversial. In this context, an increasing number of studies concluded that schools were not major transmission sites of SARS-CoV-2. For example, Ehrhardt et al. investigated the sources of infection of 557 children and adolescents aged 0–19 years after the reopening of schools and kindergartens in Baden-Württemberg (Germany) after the first lockdown in May 2020. They were able to show that infections in schools and in the context of childcare accounted for only 3.3% of all detected infections (18). Similarly, in the present study, no evidence was found that the participating facilities represented a particular risk factor for infection with SARS-CoV-2 or its onward spread. Here, the corresponding hygiene programs of the facilities in particular could have made a significant contribution to infection control.

Moreover, the study was intended to shed light on the living conditions and preventive behavior of students in DS and SBS and thereby identify age and cohort specific behavioral and attitudinal risk factors for infection via questionnaire, which was explicitly designed for the study population. Validated questionnaires from previous surveys that involved an increased risk of infection with SARS-CoV-2 in the context of the students' living conditions and activities were lacking. Thus, the questionnaire was designed mainly based on two superordinate factors in order to provide an assessment regarding the risk of infection with SARS-CoV-2: first, interpersonal contacts and second, compliance with infection-preventive hygiene measures. Further factors based on the current state of research and on reflections about everyday life from the students' point of view were included. On top, questions about COVID-19 related symptoms were implemented based on the main clinical symptoms of SARS-CoV-2 known at this time and mentioned by Huang et al. (19). In addition, a free-text option was implemented to cover any symptoms of infection with SARS-CoV-2 not yet reported in the literature at this point. The number of interpersonal contacts in the domestic environment was surveyed by asking for the number of people living in the household, as Koh et al. (16) reviewed that household transmission of SARS-CoV-2 has been identified as a significant route of infection. Although a correlation of the number of family members and the risk of infection had not been investigated until then, it was likely that a higher number of persons in the same household is accompanied with a higher number of interpersonal contacts and thus an increased risk of infection with SARS-CoV-2. Further questions were aimed at interpersonal contacts outside the families (friends, training partners, etc.). By means of questions about meetings with friends, it was intended to get an overview of the number and type of peer group contacts in the students' leisure time. In this context, it was not only the frequency of meetings that was of interest, but also the compliance with general hygiene regulations during the meetings (maintaining a minimum distance of 1.5 m and wearing a facemask) since previous studies had shown that these points had an influence on the risk of viral transmission (16, 20–24). In addition, the location of meetings and sporting activities were surveyed, as it is described in the literature that the risk of virus transmission and infection is less likely to occur outdoors than indoors (15, 23). Furthermore, a question was asked about at-risk patients in the family environment (persons over 60 years of age, persons with chronic underlying diseases, immunosuppressed persons) (8, 25), since in any cases greater prudence and compliance with hygiene regimens by students can be assumed to protect their at-risk family members. However, this had not yet been scientifically investigated. Therefore, the study participants were additionally asked about their opinions about the generally applicable hygiene guidelines to get insights into the question whether a low assessment of the usefulness of hygiene measures is associated with an increased risk of infection with SARS-CoV-2 within the cohorts. The last set of questions dealt with the use of public transportation and travel behavior, two factors that could mean an unmanageable number of interpersonal contacts and thus represent a possible additional risk factor for infection. Thus, the aim of the questionnaire was to screen symptoms and risk factors for infection and to assess whether the two cohorts differed in these aspects. Of course, the retrospective recording of the questions could have led to a recall bias. Nonetheless, important insights into cohort-specific characteristics were gained. In this context, the symptoms of illness surveyed over the study period differed only slightly between the study groups in terms of type and frequency. The decrease in symptom frequency over the study period in the SBS group is most likely explained by an increasing compliance of the athletes with hygiene measures, which could be related to the upcoming competition period in the winter months. It is also conceivable that athletes did not truthfully undertake the reporting of symptoms of illness, as this might possibly have led to quarantine-related exclusion from sport and competitions. This factor did not play a role in the DS group, which may be why the reported occurrence of disease symptoms remained rather constant in this group. Nevertheless, despite the occurrence of disease symptoms, the infection numbers with respect to SARS-CoV-2 remained very low. Of course, other pathogens may have played a role here, which were not assessed by the study design. With respect to the risk stratification for SARS-CoV-2 infections, extracurricular activities, frequency of travels, and subjective ratings of the usefulness of infection prevention measures did not highlight cohort-specific differences. Across all study participants, an overall increased risk of infection might have been expected because of poor adherence to wearing facemasks when meeting other persons, which is considered an effective infection prevention method (26–29). In addition, presumably the living conditions of the students pose a higher risk of infection compared with the general population, since school-related class communities and sports-related training groups are difficult-to-avoid circumstances in which many persons and close spatial contacts occur. In this context, the Christmas holidays, which fell within the study period, posed another unavoidable risk factor for SARS-CoV-2 infection for the students and athletes, who mostly spent the holidays with their families. The recommendations in Germany stipulated that no more than 10 people should gather for family celebrations and travel abroad was discouraged. Nevertheless, many interpersonal contacts presumably remained unavoidable during the Christmas vacations.

With regard to the risk of virus spread during sport sessions, a higher risk of infection cannot be assumed generally, since different types of sport differ significantly from each other in terms of risk factors for infection. In this context, a differentiation between individual and team sports is mandatory. Based on current research, team sports appear to have an increased risk of infection with SARS-CoV-2 compared to individual sports (30–32). The participating athletes at the sports boarding schools were all practicing individual sports, which is due to the sports profile of the sports boarding schools. Here, predominantly individual winter sports such as cross-country skiing, biathlon and ski jumping were represented and thus sports that are practiced primarily outdoors. Therefore, a comparatively lower risk of virus transmission and infection with SARS-CoV-2 could be associated in comparison with the practice of team sports, which should be considered in a comprehensive risk stratification for sports boarding schools. In this context, future investigations of differences between sport boarding schools hosting team athletes vs. sports boarding schools hosting individual athletes would represent an interesting question. However, the group comparison presented here compared a sports boarding school cohort of individual sports vs. a day school cohort that differed significantly in terms of frequency and intensity of physical activity in general. Thus, the study design does not allow comparisons between team athletes and individual athletes.

But, based on the present survey, cohort specific risk factor patterns emerged between the two study groups. For example, DS students met more often with other persons than SBS athletes. Compared to the SBS students, DS students would have an increased risk profile because they were more likely to meet people in private settings and indoor areas, less likely to consistently maintain the recommended minimum distance of 1.5 m and more likely to use public transportation. When meetings with friends occurred in the SBS group, infection prevention measures were more likely to be observed (meeting outside, keeping sufficient distance between each other) than in the DS group. This indicates an overall better compliance with preventive measures of the SBS group and suggests that there was a better awareness of the consequences of SARS-CoV-2 infections. It seems plausible that athletes are intrinsically motivated to continue their sporting activities and that this leads to greater compliance with infection prevention procedures. However, the fact that athletes tended to adhere to hygienic measures during meetings with friends could also be based on better education and the fact that an infection with consecutive quarantine and sports prohibition would have prevented the practice of competition and training activities, which can lead to serious disadvantages in competitive sports (e.g., loss of squad status, missing qualification for international competitions, etc.). On the other hand, SBS students showed an elevated risk profile for infection with SARS-CoV-2 compared to DS students because they were exposed to others without facemasks more frequently and for longer periods of time due to sports and training activities. Additionally, they traveled for longer periods of time mostly to attend competitions or training camps. In this context, the performed bubble principle of the training groups tried to avoid a higher risk of infection in sport related circumstances. But, the only SARS-CoV-2 infection that could be evaluated in this study happened in the SBS group. A closer analysis of the individual source of infection most likely suggested an infection among a meeting with out-of-sports friends. The individual assessment of the meaningfulness of the preventive measures showed that the affected athlete did not consider the measures to be very meaningful at the beginning of the study and may not have paid particular attention to the preventive measures. This individual assessment was significantly lower than the ratings of the other subjects in the group, but increased significantly after the infection occurred. The overall survey of the assessment of the meaningfulness of the measures showed that the cohorts did not differ significantly on this point. Regardless of the possibly stronger motivation of the athletes, this could probably indicate that the general educational measures of the population achieved adequate effects also for DS students as the topic had disproportionate relevance in everyone's daily life at this time. However, there could also be a selection bias that induced anyway prevention-motivated students to voluntarily participate in the study. Based on the survey results, there would nevertheless be potential for improvement in both groups regarding adherence to infection prevention measures. For example, deficits were found in adherence to the facemask regimen in the context of gatherings. In addition, according to the students' assessment, there were only a limited number of students who strictly adhered to the hygiene rules, which suggests an overall rather insufficient adherence to the hygiene rules within the facilities. Thus, it can be concluded that in the future students should be provided with enhanced information on the usefulness and necessity of compliance with facility specific infection prevention measures. If necessary, more individual and age-related educational measures could be implemented to promote understanding and even more consistent implementation of the hygiene measures (e.g., avoidance of interpersonal contact in the recreational area, consistent wearing of the facemask, adherence to the minimum distance). However, this should only be done with thorough consideration of the accompanying impact on the psychosocial situation of the students. In this context, already performed school closures and contact restrictions placed particular demands on children and adolescents. Social contacts with peers represent an important factor for young people in finding their identity, social development and general wellbeing (33–35). Negative consequences of the pandemic situation on the psychological and physical wellbeing of young people in Germany were investigated as part of the nationwide COPSY study. Decreased quality of life and health behaviors were observed. There was also a significant increase in mental health problems among children and adolescents (36). The long-term effects of the pandemic on children and adolescents and any educational gaps, that may have occurred, will not be fully understood yet. In sports boarding schools, cases of SARS-CoV-2 temporarily led to boarding school closures and training cancellations. This could lead to career disadvantages for athletes if the quarantine affected important training camps, selection games and viewings or if there were setbacks in performance due to training absences, for example. The appearance of new virus variants will probably necessitate a new evaluation of the situation and an adaptation of hygiene concepts. In the long term, efficient hygiene plans and concepts for educating students and guardians must be further developed to guarantee continuous school and boarding school operation.

However, it is important to consider that only a small number of subjects could be recruited for this study, which cannot be considered representative of an entire population. Therefore, the results can't be transferred to larger student cohorts due to the small number of cases and the single-center design. This represents an important limitation of the study, which was based on a lack of willingness to voluntarily participate in the study, especially within the day school institutions, their students or their parents. But, on an institutional level it should be mentioned that schools were particularly burdened by the pandemic situation and the need to develop and implement hygiene concepts. Furthermore, the fear of school closures and negative media image due to SARS-CoV-2 cases discovered during the study may also have played a role in the decision not to participate. Another reason for refusal could have been that day schools did not want to subject their students to more measures, whose benefits were not yet foreseeable, especially since most of the students were in their high school graduation preparations. On an individual level, low assessment of the benefits of the study or the fear of an additional time burden due to study participation could have played a role not to participate. An aversion to venous blood sampling and oropharyngeal swabs could also have led to a refusal, especially since most students were already regularly swabbed in other settings.

In conclusion, this study examined that sport boarding school students had a cohort-specific risk constellation for infection with SARS-CoV-2 due to their living circumstances. But, living in a cohort accommodation, such as the participating sports boarding schools, did not present itself as a particular risk factor for SARS-CoV-2 infection compared to attending regular day schools. In this context, the implementation, consistent adherence to and enforcement of specific infection prevention measures within student-caring institutions may ensure the continuation of school and sports operations even in pandemic situations. But, the study results cannot be generalized to other athlete facilities due to the sport profile of the institutions and the small number of study participants. Therefore, in future pandemic scenarios larger-scale, multicenter studies and larger cohorts would be desirable parameters of study designs to address these circumstances and attract more institutions to respective research questions.

## Data availability statement

The raw data supporting the conclusions of this article will be made available by the authors, without undue reservation.

## Ethics statement

The studies involving humans were approved by Research Ethics Committee, University of Freiburg, 79106 Freiburg, Germany. The studies were conducted in accordance with the local legislation and institutional requirements. Written informed consent for participation in this study was provided by the participants' legal guardians/next of kin.

## Author contributions

Conceptualization and funding acquisition: SP and PD. Methodology: VP, PD, and DH. Validation and data curation: PD and PM. Formal analysis: FB, VP, and PM. Investigation: FB, VP, OM, and OK. Resources: PD and DH. Writing—original draft preparation and visualization: FB and VP. Writing—review and editing: PM, OM, SP, and PD. Supervision and project administration: PD. All authors have read and agreed to the published version of the manuscript.
